# Factors influencing complementary feeding practices in rural and semi-urban Rwanda: a qualitative study

**DOI:** 10.1017/jns.2021.37

**Published:** 2021-06-09

**Authors:** Maryse Umugwaneza, Lize Havemann-Nel, Hester H. Vorster, Edelweiss Wentzel-Viljoen

**Affiliations:** 1 College of Medicine and Health Sciences, University of Rwanda, P.O. Box 3286, Kigali, Rwanda; 2 Centre of Excellence for Nutrition, North-West University, Private Bag X6001, Potchefstroom 2520, South Africa

**Keywords:** Children, Complementary feeding, Infants, Qualitative, Rwanda

## Abstract

The aim of the present study was to identify and describe the factors influencing feeding practices of children aged 6–23 months in Rwanda. This is a cross-sectional descriptive qualitative study. A total of ten focus group discussions were conducted separately with mothers, fathers, grandmothers and community health workers (CHWs) from five different districts in Rwanda. The discussions were recorded, transcribed verbatim, and thematically analysed using qualitative data analysis software, Atlas.ti. The study participants were mothers, fathers and grandmothers of children aged 6–23 months and CHWs in charge of child health. Caregivers’ knowledge and beliefs about the benefits of breast-feeding and timely introduction of complementary food were found to be the primary individual factors facilitating good infant and young child feeding practices. The common belief of caregivers that infants should be given liquids (thin gruel, fruit juices and meat broth) as first foods instead of semi-solid foods was a barrier to good feeding practices. The community-based nutrition education and counselling programmes were facilitators of good complementary practices at the group level. At the society level, poverty in rural agrarian households was a barrier to optimal feeding practices. The study shows that there is a need to empower caregivers with more specific guidelines, especially on complementary feeding.

## Introduction

In Rwanda, chronic malnutrition among children under 5 years of age continues to be an important public health problem. According to the latest Rwanda Demographic and Health Survey (RDHS), 38 % of under 5-year-old children were stunted in 2015. During the complementary feeding period (6–23 months), there was a dramatic increase in the prevalence of stunting, from about 10⋅5 % among children below 6 months of age to 49 % of stunted children aged 18–23 months^(1)^.

Complementary feeding practices are essential determinants of growth, health and development for infants and young children^(2,3)^. The World Health Organisation (WHO) and the United Nations’ Children's Fund (UNICEF) recommend that infants are breast-fed exclusively for the first 6 months of life^(4,5)^. At 6 months of age, breast milk is no longer enough to meet the energy and nutrient requirements of the infant^(6)^, therefore, the WHO recommends the introduction of adequate and safe complementary foods from 6 months with continued breast-feeding until the child is 24 months or older^(7)^. Complementary foods are ‘any food or liquids, whether manufactured or locally prepared, suitable as a complement to breast milk or to a breast milk substitute, fed to infants during the complementary feeding period’^(8,9)^. In Rwanda, evidence from the RDHS indicated that breast-feeding was commonly practised, with 99 % of children breast-fed at some point in time and 87 % of children under 6 months of age exclusively breast-fed. Among the infants under 6 months who were not exclusively breast-fed, 6 % were given non-milk liquids and juice, 3 % were given other milk, 2 % were given complementary foods and 1 % were given plain water only. Complementary feeding practices were sub-optimal with timely introduction of complementary feeding for only 64 % of children of 6–8 months of age. Children of 6–23 months of age receiving a minimal meal frequency were 47⋅2 % and those who achieved a minimum dietary diversity (≥4 food groups) were 30 %. Only 18 % of children 6–23 months of age achieved a minimal acceptable diet^(1)^. In a survey of 2015, complementary diets of children aged 6–23 were found insufficient in fats, vitamin C, vitamin A, iron, zinc and calcium^(10)^.

A number of factors have been identified that influence complementary feeding practices in low- and middle-income countries: the socioeconomic status of caregivers, mother's beliefs, knowledge of complementary feeding guidelines, the influence of postnatal care and the social network and lack of decision-making power in the household^(11–14)^. Those factors can be classified at individual (child, caregiver, child-caregiver dyad), group (home/family, community, work environment) and society (food system, culture, economy) levels^(12,15)^. There are limited data on the specific factors that may influence breast-feeding and complementary feeding practices in Rwanda. Therefore, the aim of the present study was to identify and describe the factors influencing feeding practices of children aged 6–23 months in Rwanda.

Better knowledge and understanding of the factors influencing complementary feeding practices is crucial for developing caregiver-friendly and effective interventions, which will improve feeding practices for children from 6 to 23 months in Rwanda and contribute to addressing the high prevalence of stunting.

## Methods

The present study is a cross-sectional descriptive qualitative study that employs the methodology of focus group discussions (FGDs) to gather opinions, beliefs and experiences about complementary feeding practices among mothers, fathers, grandmothers and community health workers (CHWs) in Rwanda. Focus groups provide an environment for participants of similar backgrounds and life situations to speak freely and openly about their experiences^(16,17)^. Although qualitative data cannot be generalised because of its sampling strategy and often the small number of participants^(17,18)^, it provides in-depth rich data, which can inform the complementary feeding interventions and stimulate further research.

### Participants’ recruitment

Data collection was done in four rural and one semi-urban purposefully selected study sites. The study sites were selected to represent views from different geographical areas in the country. A purposive recruitment of mothers, fathers and grandmothers was done by a local person (a local field worker of a non-governmental organisation, a health centre social worker or CHW). The mothers, fathers and grandmothers were eligible if they were Kinyarwanda speaking (the local language in Rwanda), were aged 18 years or older and had a child/grandchild aged 6–23 months. They were invited to participate in an FGD at a venue that was convenient and central for them, such as a community hall or the usual place of village meetings. For the recruitment of CHWs who participated in two FGDs, purposive sampling was used to involve both male and female CHWs aged 18 or older representing different villages. Separate FGDs were conducted with mothers, fathers, grandmothers and CHWs and each FGD consisted of five to seven participants.

### Data collection instruments

The FGDs were facilitated by a researcher trained in conducting FGDs. Two research assistants assisted as note takers and co-facilitators. The FGDs took place in the community where the participants lived. The discussions were conducted in Kinyarwanda. The interview guide covered the following topics: (i) breast-feeding; (ii) complementary feeding; (iii) perceptions about animal source foods in the complementary diet; (iv) sources of information about infant and young child feeding (IYCF) and (v) decision-making about IYCF.

All questions asked in the FGDs were open-ended, formulated to answer the reach the research objectives, with new questions arising from the responses given in the FGD. The FGDs sought responses to the following core questions:

1. What do you think about giving breast milk to babies?
2. Which other liquids in addition to breast milk, do you think children need before 6 months of age?
3. Why do you think it is at 6 months that complementary feeding should start?
4. Why do you think children below 2 years need complementary foods?
5. Why do you feel it is important to give food from animals to children below 2 years?
6. Why do you think that there is enough information on feeding babies available to mothers in your community? From where do you get the information on feeding babies?
7. Who has the biggest influence on mothers’ decisions and actions when feeding the baby by giving information and advice?
8. Tell us a bit more about your experience (/experiences of mothers in your community) in starting to include solid foods and other liquids.
9. Is there anything else that you would like to share about your complementary feeding experience?

The duration of each FGD was approximately 60 min. Before discussions started, the researchers prepared the venues and arranged the chairs in a circular pattern to allow easy communication between participants^(19)^. All ten FGDs were audio-recorded with participants’ consent and field notes were taken by a research assistant during the discussions^(19)^.

The first FGDs with different categories of participants (mothers, fathers, grandmothers and CHWs) were fully transcribed and analysed while the data collection was still going on. The emerging themes were explored in more depth in subsequent FGDs. By the eighth and ninth FGDs, no new themes emerged from discussions (data saturation was reached). To confirm the themes that had emerged, one more FGD was conducted beyond data saturation, resulting in a total of ten FGDs to be analysed.

### Data analysis

FGDs were transcribed verbatim in Kinyarwanda, into Microsoft Word 2010.ink. by a research assistant. The analyst (first author) checked the transcripts for quality against the original recordings and against the field notes for accuracy. The computer program Atlas.ti version 1.0.50 was used for the storage of themes and subthemes. Two analysts independently read and re-read the entire dataset to familiarise themselves with the data and identify themes and patterns^(20)^. An inductive thematic analysis^(21)^ was used for coding the transcripts. Descriptive codes^(20)^ were applied to the data, then organised into themes and refined to represent the whole dataset. An inductive approach means that the themes identified are strongly linked to the data themselves. Thematic analysis is a search for themes that emerge as being important to the description of the phenomenon. In the present study, the meaning unit of analysis was an entire phrase taken from the interviews/FGD^(22)^. The meaning units were labelled with codes that were sorted and collated into themes. A theme is a group of codes that captures something important about the data in relation to the research question^(21)^. Codes and themes were reviewed and discussed by the authors. The themes were classified as individual, group and society factors influencing feeding practices of children aged 6–23 months in Rwanda^(12,15)^. The final set of themes and illustrative quotes from participants were reported.

Quotes are tagged by participant category (Mothers, Fathers, Grandmothers and CHWs) and by the district of residence (Nyamasheke, Nyaruguru, Kamonyi, Gicumbi and Gatsibo).

### Ethics considerations

Ethics approval for the present study was granted by the Ethics Committee of the NWU (NWU-00098-14-S1) and Rwanda National Ethics Committee (0251/RNEC/2015). Permission to conduct the study was granted by the Ministry of Local Governance, Rwanda.

The researchers met with local government leaders to explain the purpose of the study. The purpose of the study and the procedures were also explained to the research participants, who were given the opportunity to ask any questions they had before signing the consent forms. Illiterate participants put a thumb print on the informed consent form. Participation was voluntary. The participants were told that they could withdraw from the study at any time without any consequences for them.

## Results

### Socio-demographic characteristics of the participants

The total number of participants in the ten FGDs was sixty-five participants comprising thirty-four mothers, twelve fathers, seven grandmothers and twelve CHWs. We conducted five FGDs with mothers, two FGDs with fathers, one FGD with grandmothers and two FGDs with CHWs. Table 1 shows the socio-demographic characteristics of the participants.

**Table 1.** Socio-demographic characteristics of the study participants (*n* 65)

	Number	Percentage
Age range (years)		
<30	12	18
30–49	40	62
>50	13	20
Education level attained		
Never attended school	6	9
Primary school 1–3	6	9
Primary school 4–6	28	43
Secondary school	20	31
Tertiary and professional education	5	8
Occupation		
None	6	9
Farmer	50	77
Informal trading	2	3
Formal employment	7	11

### Overview of the factors influencing feeding practices in rural and semi-urban Rwanda

A set of factors influencing feeding practices in rural Rwanda emerged from the data. Those factors are classified as facilitators of/barriers to adequate complementary feeding practices at the individual, group and society levels (Table 2).

**Table 2.** Overview of factors influencing caregivers’ complementary feeding practices in Rwanda

Facilitators	Barriers
Individual level	
Knowledge about breast-feeding and complementary feeding	Maternal beliefs about breast milk production and infant hunger cues Beliefs about appropriate complementary foods Availability and affordability of complementary foods
Group level	
Information and counselling about child feeding	Dependence of the mother on the father's financial support Burden of other household chores and farming activities by the caregiver
Society level	
	Poverty

#### Individual level factors

##### Knowledge about breast-feeding and complementary feeding

In general, participants reported better general health for the baby, better growth and cognitive development, and family planning, as advantages of breast-feeding. The following quotes illustrate participants’ understanding:

‘*When you continue breastfeeding after six months, the child grows well, gets intelligent and gets good stature. It's also after six months that you start to give him/her fruits, that helps the child because breast milk does not fill him/her anymore’ Mother, Kamonyi*

*‘In my understanding, breastmilk contains all nutrients that a child's life needs’ Father, Nyamasheke*

*‘Breastfeeding helps the child; it gives the vitamins to nourish his/her life’ Grandmother, Nyaruguru*

The discussions indicated that participants associated the introduction of complementary foods at 6 months of age with the physiological maturation of the infant's stomach. They believed that the reason why it is recommended to start complementary feeding at 6 months is that babies have a ‘bigger’ and/or ‘stronger’ stomach.

‘*As the child grows, his/her stomach becomes bigger so he/she needs complementary foods to feel full’ Father, Nyamasheke*

Participants associated early introduction of complementary food with poor growth and frequent illnesses, as illustrated by the following quotes:

‘*When you give food early it causes poor health to the child, but when you start complementary feeding at six months the child grows well and does not lose his/her good health’ Mother, Gatsibo*

##### Maternal beliefs about breast milk production and infant hunger cues

Some participants reported that the introduction of complementary foods at 4 or 5 months was sometimes practised. The main reason cited for the early introduction of complementary foods was perceived insufficient production of breast milk. Participants also said that insufficient production of breast milk was caused by insufficient food intake.

‘*In such a case of a child who would not get full with breast milk, usually at around four months they start giving him/her gruel’ Mother, Nyamasheke*

Gruels are watery cereal and legume-based porridges.

Mothers said infant hunger cues, such as the baby wanting to touch food/table utensils when others were eating, led to the introduction of complementary foods.

‘*At around four months, when you take a cup, the child reaches out to catch the cup because he/she is hungry. I mix gruel with milk and I give him/her’ Mother, Kamonyi*

##### Beliefs about appropriate complementary foods

The consistency of the complementary food, especially at the beginning of complementary feeding, was very important for participants, and the most important characteristic of an acceptable complementary food. With respect to the consistency of foods, the participants’ concerns were about the physiological maturation of the stomach to a great extent, and of the dentition to a lesser extent. Participants reported practices such as sieving flour or straining cooked gruel to make it as fine as possible. Homemade fruit juices, fruit puree and thin gruel were mentioned by caregivers to have a consistency that is adequate as first foods. In general, most participants reported that liquids (gruel, milk, fruit juice, meat broth) are first introduced. Then, solid foods (potatoes, banana, etc.) are mashed into a puree for children aged approximately 8 months and beyond. However, gruel was said to remain the main ‘drink’ for children.

‘*Gruel is something that is affordable, and parents think the child has a soft stomach that would not handle other foods’ CHW, Gicumbi*

*‘When my child was six months old, I went to the market and bought fruit, I came home and took tree tomatoes, passion fruit and oranges, and I used a tea strainer to make juice. Because he couldn't eat fruits, I first gave him/her fruit juice. After that I bought fish, boiled it and give my baby the fine broth. Those were the first complementary foods’ Mother, Kamonyi*

Most participants said that meat is suitable when the child is 9 months old or older. They believed that is when the child's teeth and stomach can handle the texture of meat. A few participants said that they used meat/fish broth in their infants’ and young children's food.

‘*… we use small dried fish instead; we pound it so that children, who cannot chew, eat it with vegetables’ Grandmother, Nyaruguru*

Besides, several participants said the capacity of foods to fill the stomach was important to them. Some mothers mentioned that they give foods such as eggs, fish and fruits but because those foods were not ‘filling’ the child, the mothers would switch to bulkier feeds such as gruel.

‘*When my child started eating his father bought him/her an egg and a banana, but the child was not getting full and was not gaining weight. So, I bought sorghum and soy flours to make porridge for him/her. Now he/she is better’ Mother, Nyaruguru*

Some foods were found to be avoided during the complementary feeding period. Some participants said that dried beans are unsuitable for the young child. They believed that dried beans cause diarrhoea to the child.

‘*People say beans cause diarrhoea to children, that one should not give beans to a child’ Mother, Nyaruguru*

*‘Parents give beans to children usually after one year and above because before that age they would need to remove the beans’ skins’ CHW, Nyamasheke*

However, most participants said that they give beans to their children, emphasising the need to add vegetables to the beans and mash them.

‘*I mix beans and vegetables, then I mash all of it to make it soft for the child’ Mother, Gicumbi*

When discussing how they prepare food for the child, some participants said that they avoided using oil in the food served to children.

‘*To avoid putting oil in the child's food, at the beginning of complementary feeding, you cook a separate pot for the child’ Mother, Kamonyi*

*‘I boil potatoes with vegetables, and when it's cooked, as I am about to put oil, I first put aside some food for the child’ Mother, Gatsibo*

##### Availability and affordability of complementary foods

Most of the participants said that they give gruel because that is what they can afford, and many mentioned that they would give cow milk instead if they could afford it. The first challenge to appropriate complementary feeding noted by participants in all FGDs is the affordability of food from the markets. Small dried fish were the animal source food that the majority of participants considered most affordable, physically available in all areas and adequate for infants.

‘*Most of the time milk is for those who have means, but some people in rural areas make gruel, they say that the mother does not have breast milk so they give gruel to the baby because that is what is within their means’ Father, Nyamaskeke*

*‘Usually when a child starts eating, when you don't have milk, you take soy beans, maize and sorghum grains to the mill, and you give gruel to your child’ Mother, Nyaruguru*

Food produce grown by the household was more likely to be used as complementary food.

‘*The reason why we choose gruel is because it is easier for us farmers, when you don't have a cow, there is nothing else you can use apart from gruel and soy milk’ Mother, Gatsibo*.

However, in one focus group, mothers discussed the frequency of buying biscuits for their children.

‘*… sometimes you end up buying biscuits every day, possibly spending about 700 Rwandan francs every week’. Mother, Kamonyi*

The mentioned biscuits are usually sweet plain unfortified biscuits. The amount of 700 Rwandan francs was equivalent to about one USD at the time the FGDs were conducted.

#### Group level factors

##### Information and counselling about child feeding

In all discussions, the caregivers (fathers, mothers and grandmothers) reported CHWs to be their first source of information. In Rwanda, CHWs are lay people trained to educate and counsel the community they live in on major public health issues. Community-based programmes; local leaders and churches were also cited as sources of IYCF information.

CHWs were perceived as the most important sources of information because they live in the community; they were accessible and could give individualised counselling. The monthly community work session (umuganda) and community cooking sessions were also mentioned in all focus groups as a platform where they got information on IYCF from CHWs. Parents and grandmothers, in general, expressed great confidence in the information provided by CHWs. The use of images on education materials was perceived as compelling by CHWs and mothers.

‘*Most of us parent listen to community health workers’ teachings. They teach us in our households, and the teachings they give us are accompanied by images. They show you, discuss with you how it works, and when you hear announcements on the radio, you find that the radio is repeating what community health workers were saying’ Mother, Nyamasheke*

Village Kitchen (Igikoni cy'umudugudu) is a community programme where caregivers meet on a weekly or monthly basis to cook a meal for their children and to be educated by the CHWs. Most mothers perceived Village Kitchen as a source of knowledge and skills to prepare complementary foods. They feel more confident about giving children the food they have prepared before in the Village Kitchen sessions.

‘*We also have the village kitchen. Everyone goes there with food from their house and they give us oil, sometimes small fish too. The children eat, and when there are leftovers we eat too’ Grandmother, Nyaruguru*

*‘We also get information from the Village Kitchen. …when you feel like things are not clear, you go there and they explain many things to you’ Father, Gatsibo*.

In the FGDs with fathers, some fathers said that their wives were also an important source of information about IYCF, as the mothers get the information from antenatal consultations and education sessions during vaccination campaigns, where some fathers do not go.

‘*When our wives go to antenatal sessions, they don't keep the information to themselves, they tell us what they learned so that we help each other raise the child when he/she is born’ Father, Gatsibo*

Health centres’ pre- and postnatal education sessions were also discussed as sources of information, mainly for mothers.

‘*Even before the community-based groups were put in place, there were health centres, as you know when a woman is pregnant, they follow her until she gives birth, and they monitor the baby and immunise him/her. One gets information from there too, because of the education sessions before immunisation’ Mother, Nyaruguru*

In addition, radio was also mentioned by participants as a source of information.

‘*Men sometimes don't go with women for the child vaccinations but even on the radio we can hear and learn things’ Father, Nyaruguru*

##### Dependence of the mother on the father's financial support

In all discussions, participants said that the primary role of fathers in IYCF was to provide money; however, some participants reported that a dialogue took place between the two parents about the child's food. Fathers reported that they were willing to contribute to the day-to-day caregiving of the child when the mother was ill or temporarily unavailable.

‘*Women are the ones who are able to prepare what children need. As men, our role in the nutrition of children is to provide means to buy what is needed’ Father, Nyamasheke*

*‘The father knows how to provide food for the household. When it is provided, I must prepare the meal for the child with what was provided’ Mother, Gatsibo*

##### Burden of other household chores and farming activities

Some participants perceived continued breast-feeding, complementary feeding and caring as competing with agricultural and household chores.

‘*Mothers have a lot of chores, so they leave the babies with their older siblings because of the work, and if the mother has prepared food for the baby the older ones might eat it’ CHW, Gicumbi*

*‘When they first start complementary feeding at six months, most mothers do their best, problems start when the child can be left at home, they still need breast milk and complementary foods, but because the mother sees that the child can now eat they somehow become less attentive’ CHW, Gicumbi*

#### Society level factors

##### Poverty

Financial constraints were cited as an important barrier to complementary feeding.

‘*Poverty is the main challenge we have. Because if we had means, there would be no other reason not to give our children an adequate complementary diet’ Mother, Nyamaskeke*

## Discussion

The present study extends the qualitative literature on IYCF by exploring the factors influencing child feeding practices in a sample of mothers, fathers, grandmothers and CHWs in Rwanda.

In the present study, participants discourse showed how they associate following the official breast-feeding and complementary feeding guidelines with good health and growth for their children. However, mother's perceived breast milk insufficiency and perceived child hunger/readiness for solid foods were barriers to exclusive breast-feeding for the first 6 months of life. Both barriers have been previously reported in the literature^(14,23,24)^.

The belief that infants of 6–8 months cannot eat semi-solid food was also an important barrier to optimal complementary feeding. Most caregivers served thin gruels to children as a staple. Thin gruels are very common first foods in sub-Saharan Africa^(25,26)^. Because of the high quantity of water and low quantity of flour used in their preparation, gruels are low in nutrients and energy^(27)^. The present study points out that the consistency of complementary food, especially at the beginning of complementary feeding, is of great concern for caregivers who perceive the need to first give feeds of liquid consistency.

In the present study, caregivers’ narratives emphasise the role played by CHWs in nutrition education in Rwanda. The findings show that caregivers, in general, received information and counselling from the CHWs through community-based nutrition education platforms such as the Village Kitchen programme. This reinforces findings from other studies indicating that CHWs play an important role in interventions for child survival in community settings^(28–30)^.

Rural agriculturalist mothers have a heavy burden of field work and household chores (preparing food for all household members, fetching water, etc.) to perform daily. Time allocation to different chores was identified as a barrier to adequate feeding and care in other countries as well^(31,32)^. In accordance with the review of Aubel on the wider household members’ roles in child nutrition^(33)^, our findings also show that in Rwanda, mothers performed the day-to-day nutrition activities, while fathers were less involved.

Grandmothers and fathers were knowledgeable about the benefits of optimal breast-feeding practices. This supports the need to continue educating all community members through community platforms such as ‘umuganda’ (monthly community work) and other mass media. Moreover, in a quasi-experimental study performed in Kenya, improved knowledge of fathers and grandmothers on health and nutrition of infants and young children resulted in improved social support reported by mothers in terms of physical action and material support^(34)^.

Caregivers reported poverty as a barrier that affected their feeding practices. Even though most of them were agriculturalists, they explained that it was difficult for them to afford complementary foods. This may be explained by the fact that in Rwanda crop-growing households do not produce enough to cover their food needs; about 70 % of household food is bought at the market, while only a quarter comes from households’ own production^(35)^. Household food insecurity in agriculturalist communities was found by Burns *et al.* in the Democratic Republic of Congo as well^(14)^. Nutrition education could help mitigate apparently low access to nutritious food. For example, the mothers in one focus group estimated that they could spend up to RWF 700 every week to buy biscuits for children. The RWF 700 estimated by mothers was equivalent to about one USD (using the exchange rate of the year 2015). The same amount could, for example, buy seven eggs or about 250 g of meat (prices of 2015). It is possible that perceptions of biscuits being nutritious and convenient may be a motivating factor for caregivers to buy those foods. Contrary to this perception however, studies have shown that processed high sugar foods may increase the risk of obesity and metabolic risk^(36)^.

The present study shows that the factors influencing child feeding practices in a sample of rural caregivers in Rwanda are at the individual, group and society levels.

### Strengths and limitations

The present study had a number of limitations, the first of which was the lack of methodological triangulation: the methodology did not foresee any in-depth interviews or observations. Secondly, the focal persons used to recruit participants for the study were linked to community nutrition education. This may have introduced a social desirability bias where participants would have answered what they assumed was the right thing to do rather than what they actually did.

In spite of the above-mentioned limitations, the results fit well within the current scientific knowledge reported from other countries in sub-Saharan Africa, and we are confident that the above limitations did not greatly reduce the validity of our findings.

The strengths of the study are that the participants were recruited from a range of locations and varied in terms of age, education level and occupation; therefore, it seems likely that a broad range of views and experiences were elicited.
